# 2D BeP_2_ monolayer: investigation of electronic and optical properties by driven modulated strain

**DOI:** 10.1039/d0ra03599h

**Published:** 2020-07-17

**Authors:** Shivam Kansara, Yogesh Sonvane, P. N. Gajjar, Sanjeev K. Gupta

**Affiliations:** Department of Physics, SMMPISR Kadi Sarva Vishwavidyalaya Gandhinagar 382015 India shivam_msc@ldrp.ac.in; Advanced Materials Lab, Department of Applied Physics, S.V. National Institute of Technology Surat 395007 India yas@phy.svnit.ac.in; Department of Physics, Gujarat University Ahmedabad 380009 India; Computational Materials and Nanoscience Group, Department of Physics, St. Xavier's College Ahmedabad 380009 India sanjeev.gupta@sxca.edu.in

## Abstract

Recently, the two-dimensional (2D) material beryllium diphosphide (BeP_2_) has attracted significant attention for potential device applications due to its Dirac semimetal state, dynamic and thermal stability, and high carrier mobility. In this work, we investigated its electronic and optical properties under biaxial Lagrangian strain using density functional theory (DFT). Electronic band gaps and effective charge carrier mass were highly sensitive to the Lagrangian strain of BeP_2_ monolayer. The bandgaps of BeP_2_ varied from 0 eV to 0.30 eV for 2% to 8% strain, where the strain range is based on the final stable condition of the system. The absorption spectra for the dielectric properties show the highest absorption peaks in the infrared (IR) region. These abundant strain-dependent studies of the BeP_2_ monolayer provide guidelines for its application in infrared sensors and devices.

## Abstract 2

BeP_2_ monolayer is a promising material for the novel IR optical device.

## Graphical abstract

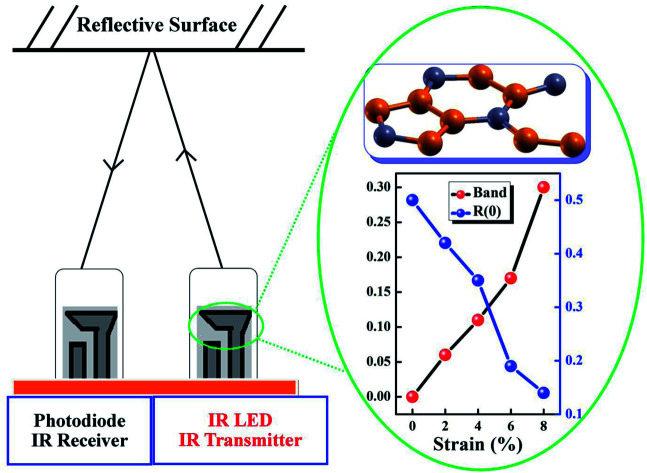

## Introduction

Strain effects in systems (metal, semiconductor, and insulator) are considered to be highly sensitive effective and convenient tools for their tuning electronic, transport, and optical properties.^1–6^ The applied strain effects can be interpreted as an elastic field applied to materials, which modifies the geometrical structure of their crystals due to the interaction between the elastic field and crystalline field, thus influencing the electronic band structure,^7^ and finally tuning the physical,^8^ chemical and catalytic properties^9^ of materials. These tools are especially reasonable for the design of two-dimensional (2D) crystals because their low-dimensional structure can sustain much larger strain compared to bulk crystals.^10,11^ For example, the black phosphorus (BP) monolayer has been strained up to a noteworthy value of 30% without any dislocation or plastic deformation in its crystal structure,^12^ giving a wide range to tune its mechanical and electronic properties.^1^ Besides, as a universal structure, the 2D BeP_2_ material with Dirac cones found precisely at the Fermi level,^13^ high carrier mobility and novel strain-tunable Dirac semimetal state of ultrathin BeP_2_ can also be altered by strain.

Beryllium di-phosphide (BeP_2_) has attracted significant attention recently due to its Dirac semimetal state, excellent stability in the ambient environment, thermal stability, and high carrier mobility. Herein, we concentrated on a P and Be atomic sheet because Be is generally utilized as one of the constituent components in 2D binary compounds, including h-BeS,^14^ Be_2_C,^15^ and Be_5_C_2_.^16^ Besides, a BeN_2_ nanosheet is considered to be stable with outstanding properties due to its high stability and direct bandgap.^13,17^ Dirac semimetals are incredible directors of power, even though they have zero band gaps, and thus cannot be turned off. Due to the specific limitation of topology insulators in photonic devices,^18,19^ we applied Lagrangian strain to the BeP_2_ monolayer for bandgap opening. To date, there are no reports on engineering the physical and corresponding anisotropic properties of BeP_2_, and thus the present work focused on the examination of its in-plane anisotropic properties, including its stable structural, electrical, optical and anisotropies.

Nowadays, various researchers have focused on Dirac semimetal materials due to their rapid charge transport and significantly high carrier mobility.^20–25^ There are numerous reports on tuning the electronic properties of Dirac semimetal, semiconductor, and insulator monolayers for functional devices.^20,23,26–36^ Wang *et al.*^26,27^ reported a tunable 1T-WTe_2_ monolayer semimetal having a small bandgap by ambipolar conduction. Yu *et al.*^29^ reported tuning the bandgap of bilayer graphene *via* molecular doping with benzyl viologen molecules. Semi-metallic single-layer buckled silicene and germanene were transformed into an open bandgap using an external electric field.^31^ Mu *et al.* reported a metallic to semimetal transition with a Dirac point in BeB_2_ monolayer *via* 5% isotropic compression, while it became metallic again under a larger compression.^20^

In this work, the effects of strain on monolayer BeP_2_ were investigated using first-principles calculations. We investigated the change in the electronic and optical properties of the BeP_2_ monolayer under Lagrangian strain. Bandgap engineering using strain is an effective method and has been used in other 2D thin films. Besides, the strain effects on the effective mass of electrons and holes were deliberately explored.

## Methodology

**Fig. 1:**
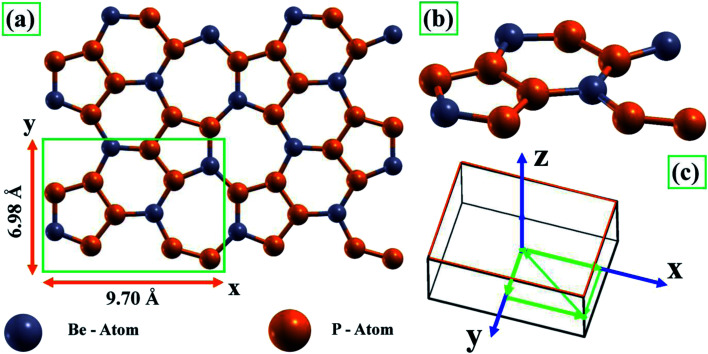
(a) Top and (b) side views of BeP_2_ monolayer. (a) The top view represents the 2 × 2 supercells of the BeP_2_ monolayer and lattice constant of the unit cell (green box). (b) Represents the diagonal view of the BeP_2_ monolayer (unit cell). (c) Corresponding Brillouin zone for the orthorhombic structure.

We have carried out *ab initio* DFT calculations, which were implemented using the Vienna *Ab initio* Simulation Package (VASP).^37,38^ The projector augmented wave (PAW)^39^ pseudopotential with the Perdew–Burke–Ernzerhof (PBE) exchange–correlation function was exploited to study the electronic properties of the semiconductor BeP_2_ monolayer. An orthorhombic unit cell was adopted for the planer global structure (shown in Fig. 1). Here, a vacuum spacing of greater than 16 Å was employed to prevent mirror interactions with the imaginary system in the direction perpendicular (*z*-axis) to the nanosheet. For the plane-wave basis set, we considered a kinetic energy cut-off of 500 eV. All structures were fully relaxed up to ionic forces less than 0.001 eV Å^−1^ for each atom using the conjugate gradient method from the Hellmann–Feynman theorem. The Brillouin zone (BZ) integrations were sampled using 10 × 10 × 1 and 12 × 12 × 1 special *k*-point sampling in the Monkhorst–Pack scheme^40^ for the geometry optimizations, and electronic and optical properties, respectively. The convergence criteria for the energy cut-off, *k*-point mesh, and smearing width were performed to reach an accuracy of total energy error less than 0.001 eV per cell. To investigate the strain effects on the atomic configuration and the electronic structure of the monolayer BeP_2_, we applied an external strain to the biaxial xy-axis, and allowed all atoms to freely relax. To calculate the dielectric frequency-dependent optical properties such as dielectric function, electron energy loss spectra (EELS), and reflectance and absorbance spectra, the random phase approximation (RPA) was used.^41^

## Results and discussion

### Structure and stability

Initially, we investigated the general structure of BeP_2_ to understand its properties, which are usually determined by its structure, and then studied the effect of Lagrangian strain on the structure of the BeP_2_ monolayer. As shown in Fig. 1(a), the supercell 2 × 2 of the BeP_2_ monolayer was represented by a hexagonal shape with Be and P atoms, which is consistent with other works.^13^ One unit cell of the BeP_2_ monolayer (sketched green box in Fig. 1(a)) contains four Be atoms and eight P atoms, with a calculated lattice constant (displayed in Fig. 1(a)) of 9.70 Å and 6.98 Å for the *x* and *y*-direction, respectively. Fig. 1(b) shows the side phase of the BeP_2_ monolayer. The corresponding path through the high symmetry points in the first Brillouin zone for the orthorhombic structure due to the electronic properties is shown in Fig. 1(c). Here, one Be atom forms a trigonal bond to an adjacent N with sp^2^ hybridization and no distortion. The distance between the Be and P atoms is ∼2.06 Å (varying) and P and P atoms is ∼2.09 Å (varying), as shown by in Fig. 1, which is in good agreement with a previous work.^13^ The explanation for the stability of the ideal BeP_2_ monolayer has been reported previously based on phonon spectra.^13^

**Fig. 2:**
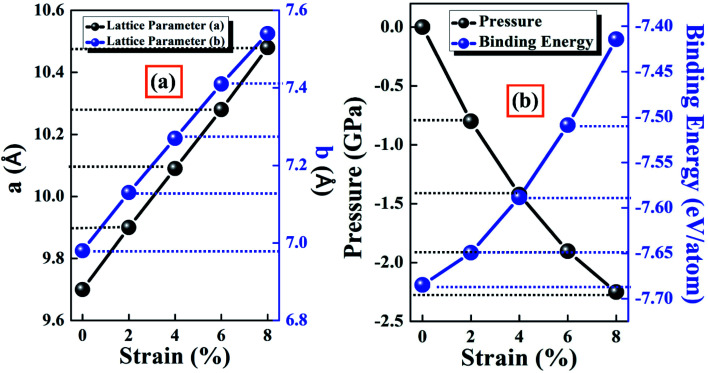
Representation of (a) lattice constants (*x*- and *y*-directions) and (b) system pressure, and binding energy per atom with various Lagrangian strain.

Fig. 2(a) presents the lattice constants along the *x*- and *y*-axis with different strain, which increased with an increase in strain. The variation lattice parameters affected the pressure, total energy, and binding energy of the system, which are responsible for the stability of the structure. Before investigating the various properties of the BeP_2_ monolayer for its potential applications, we needed to confirm its stability and feasibility for its experimental synthesis. Thus, we calculated the cohesive energy of free and strain-dependent BeP_2_ monolayers, as shown in Fig. 2(b). The binding energy *E*_binding_ can be calculated as follows:

1 *E*_binding_ = *E*_BeP_2__ − *n*(*E*_Be_ − *E*_P_)

where *E*_BeP_2__, *E*_Be_ and *E*_P_ are the total energy of the complex system, a single Be atom, and a single P atom, respectively. *n* is the number of atoms per unit cell. The binding energy of the BeP_2_ monolayer is much better compared to that of other monolayers such as graphene,^42^ phosphorene,^12^ carbon phosphide,^43^ Al_2_C sheet,^44^ silicene,^45^ and antimonene^46,47^ of 7.62, 3.48, 5.32, 3.94, 3.55 and 4.26 eV per atom, respectively. For comparison, the cohesive energy of the BeP_2_ monolayer is 7.68 eV per atom, which is larger than that of available AB_2_-type 2D materials such as CaSb_2_,^48^ BeN_2_,^17^ and MgP_2_.^49^ The high binding energy of the BeP_2_ monolayer of 7.68 eV per atom justifies its robust nature and relative stability. Upon applying Lagrangian strain, the cohesive energy is reduced with an increase in the pressure of the system. The previous study by Lee *et al.*^50^ and Corsetti *et al.*^51^ reported that systems were considered to be stable up to a pressure of ∼1 GPa for a 5 Å system, whereas in our study, we employed an ∼10 Å system, and thus the stability was sustained up to a pressure of ∼2 GPa. Thus, this proved that the system was stable under Lagrangian strain. The reported pressure is dependent on both the confinement width and the form of the confining potential. Also, a pressure of ∼1 GPa led to a slight increase in the thermal equilibrium concentration and diffusion of vacancies, but this increase was much smaller than that of self-interstitials.

### Electronic properties

**Fig. 3:**
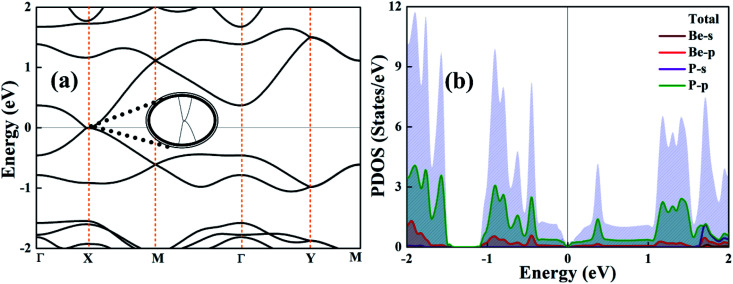
(a) Electronic band structure and (b) partial density of states (PDOS) of the BeP_2_ monolayer. The inset figure shows the intersection of the bands.

Fig. 3(a) and (b) presents the calculated band structure and partial density of states (PDOS) of a strain-free BeP_2_ monolayer. The curvature of the band structure of the BeP_2_ monolayer (Fig. 3(a)) is similar to the expected band structure of graphene, where in particular, it shows a linear Dirac-type dispersion of electrons near the *X* points. However, the Fermi velocity, *ν*, of the electrons in the BeP_2_ monolayer is lower than that of graphene.^52^ The PDOS, as shown in Fig. 3(b), demonstrates the contribution of the orbitals of the atoms. There is a larger orbital contribution from the p orbital of the atoms than the s orbital. However, the p orbital of the P atom has a larger contribution than the p orbital of the Be atom with zero density of states (DOS) at the Fermi energy, which is responsible for the semi-metallic behavior. The presented electronic properties are in very good agreement with that previously reported by Li *et al.*^13^ They have presented the orbital projected band structure, which can be used to evaluate the orbit correlation and is responsible for the semi-metallic behavior.

**Fig. 4:**
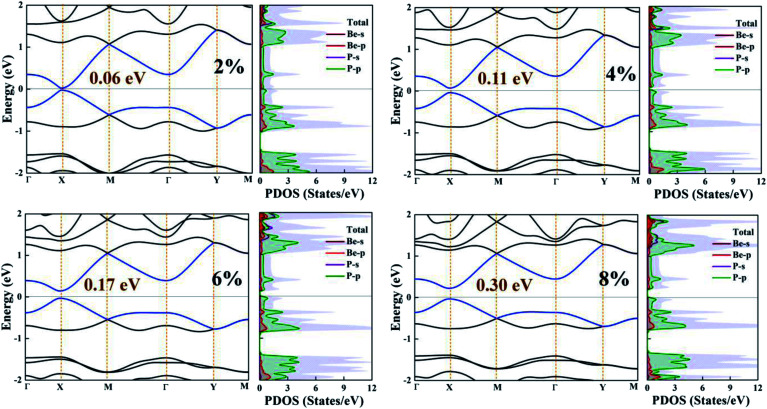
Electronic band structure and partial density of states (PDOS) of the BeP_2_ monolayer with various Lagrangian strain.

Here, the electronic band structure and PDOS of the BeP_2_ monolayer under Lagrangian biaxial strain is presented in Fig. 4. For biaxial strain, the strain tensor (*e*) can be written as:2
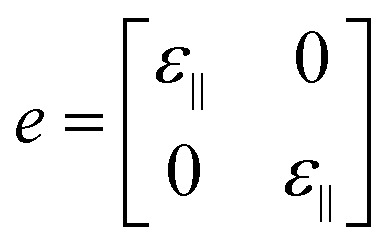
where *ε*_∥_ = (*a* − *a*_0_)/*a*_0_, *a* represents the lattice constant and the biaxial strain tensor (*e*) is implemented in the supercells by applying the changes to the lattice parameters along the *x*–*y* plane. In the biaxial strain, the varying value of *E*_X_ corresponds to the changes in strain, while there is a slight change in the values of *E*_M_ to *E*_X_. For tensile strain, *E*_X_ increases as the strain increases, with the descending rate determined to be 60 meV per 2% strain.

**Fig. 5:**
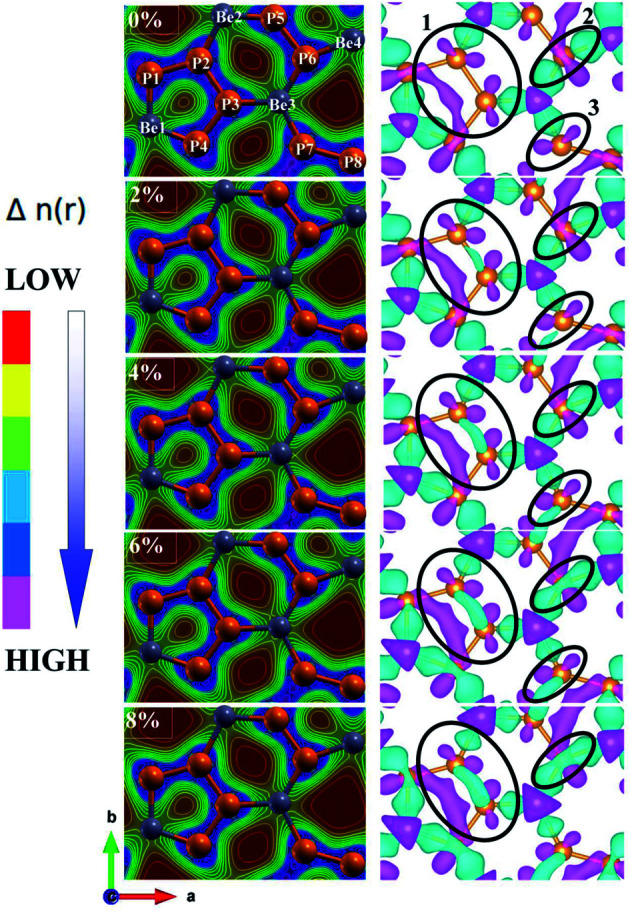
Right and left side of the electronic charge contour and electron density difference of BeP_2_ monolayer with various Lagrangian strain.

The effect of strain on the band structures of the BeP_2_ monolayer is shown in Fig. 4, which was used to calculate the values of the effective masses (electron and hole), corresponding to the valence and conduction bands. The strain effect illustrates the major changes in the electronic band structure at the *X*-point. Thus, we can say that the conduction channel with the lowest energy and valence channel with the highest energy are located at the *X*-point. Direct band gaps appear at the *X*-point with an increase in strain, as shown in Fig. 4. Insight into the bonding character of the given monolayer was derived from the electronic charge density analysis, as shown in Fig. 5. It is clear that along the atomic sphere, the electrons are not limited, and their reasonable proportion resides in the interstitial region, thus the nature of the bonding is covalent for free strain systems. We calculated the partial density of states (PDOS) of the Be atoms and P atoms to understand their orbital contribution to the band structure. Here, the valence band mainly originates (larger contribution) from the 2p and 3p states of the Be and P atoms rather than the 2s and 3s states of the BeP_2_ monolayer.

Here, the 3p states of the P atoms form a tetrahedral contribution with the 2s states of the Be atoms. The orbital states of Be-2s and P-3p have higher orbital contributions, which are demonstrated by the higher binding energies, as shown in Fig. 2(b). The conduction and valence energy bands (*i.e.* CBE and VBE, respectively) shift toward a lower energy with Lagrangian tensile strain. Due to the Lagrangian strain, the band shift at the *X*-point corresponds to the bonding and delocalization character of the CBE and VBE orbitals, respectively. Thus, it can be concluded that Lagrangian tensile strain can successfully tune the bandgap of the BeP_2_ monolayer for its wide application.

We calculated the effective masses of the electrons and holes for the band structure of the BeP_2_ monolayer using eqn (3) as follows:3
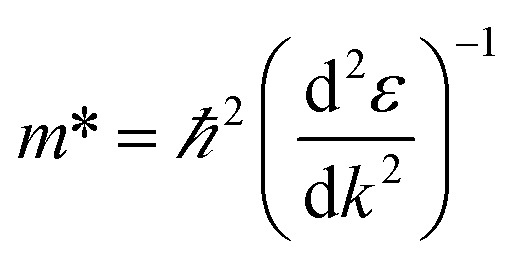


For this calculation, we used a small energy range between −0.2 eV to +0.2 eV for the conduction and valence bands near the *X*-point (bandgap), where the unit of the considered range ±0.2 is 2π/*a* (*a* describes the axial lattice constant). Here, the displayed band structure curve of energy *versus* K was fitted using the second-order polynomial *ε* = *C*_1_*k*^2^ + *C*_2_*k* + *C*_3_. According to the fitting function, we obtained the curvature 
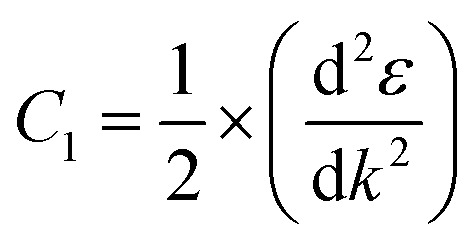
. Using the above equation and discussion, we calculated the value of the effective mass of the electrons and holes through the relation m* = *ℏ*^2^/2*C*_1_.^53^ The values of the effective mass of the electrons 
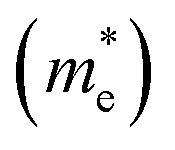
 and holes 
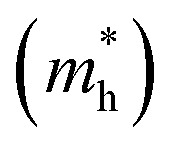
 are tabulated in Table 1.

**Table 1:** The hole (*μ*_h_) and electron (*μ*_e_) effective mass of the BeP_2_ monolayer with various Lagrangian strain for the *X*-point of the electronic band structures

Strain (%)	0	2	4	6	8
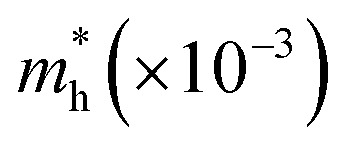	—	0.61	0.75	0.93	1.15
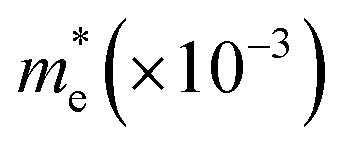	—	−0.86	−0.95	−1.35	−1.44

The energy density represents the strength or nature of bonds. The chemical bond is a concept for realizing the properties of a structure such as stability and reactivity for host materials. The calculated charge density distribution provides significant details regarding local energy density properties.^54^ The kinetic energy is dominant due to the positive Laplacian electron density, which is fundamental for the depletion of the bond charge. Thus, to gain deeper insight into the deformation of the BeP_2_ structure under Lagrangian strain, we focused on analyzing and expressing the electronic charge contour and charge difference plots in the (110) crystallographic plane, as presented in Fig. 5. The electronic charge density contour depicts the partial ionic and strong covalent bonding of the P–P and P–Be atoms based on the Pauling electronegativity difference. The atomic bonding can be observed by the color charge scale, as displayed on the right side in Fig. 5. The color charge scale represents various colors from red to pink as low electron density to high electron density, respectively. With the approach of strain, electrons are transferred from the Be to P atoms through the closest atoms. Thus, to investigate the charge transfer between two atoms of the BeP_2_ monolayer, the charge density difference Δ*ρ* was calculated, as illustrated in Fig. 5, using the following equation:

4 Δ*ρ* = *ρ*_complex_ − *ρ*_Be_ − *ρ*_P_

where *ρ*_complex_ is the charge density of the complex system of the BeP_2_ monolayer, and *ρ*_Be_ and *ρ*_P_ are the charge densities of the Be and P atoms, respectively. The cyan and pink clouds in Fig. 5 represent electron accumulation and depletion, respectively, which correspond to a gain and depletion in electronic charge, respectively. A small amount of electronic charge is depleted from the P atoms. Therefore, the black circles labeled as 1, 2 and 3 in the CDDs represent the deformation in the BeP_2_ structure upon the application of Lagrangian strain. Here, the charge transfer between the atoms can be observed. The charge density at points 1, 2 and 3 slowly increase with an increase in strain up to the ultimate tensile strength, *i.e.* 8% elongation. Beyond this strain, the BeP_2_ monolayer is observed to be a higher value than 2 GPa. Therefore, a stress–strain curve for up to 8% applied strain was observed.

### Optical properties

**Fig. 6:**
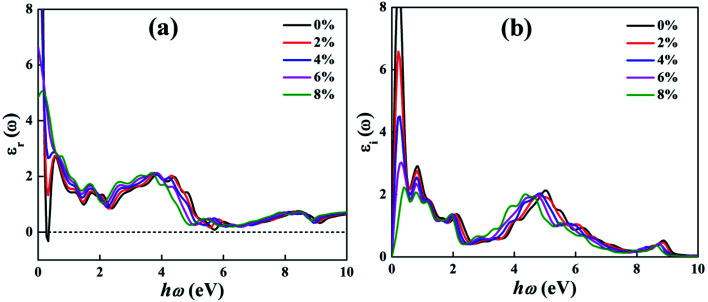
Calculated optical (a) real and (b) imaginary parts of the dielectric function of the BeP_2_ monolayer with various Lagrangian strain.

Here, we presented the imaginary and real part of the frequency-dependent complex dielectric function for the BeP_2_ monolayer under Lagrangian strain. Investigating the optical properties of the BeP_2_ monolayer is important for its optoelectronic applications. The electronic band structure of materials is closely correlated to the imaginary part *ε*_i_(*ω*), which can be represented as shown in Fig. 6(b). The transition of the interband in *ε*_i_(*ω*) occurs from the valence band maximum (VBM) to the conduction band minimum (CBM) along the *Γ*–*X* direction and vicinity of the *M*-point of all the peaks. All the optical interband transitions are essentially due to the p orbital of the Be and P atoms, as shown be the PDOS in Fig. 3 and 4. Meanwhile, the real part *ε*_r_(*ω*) represents the electronic polarizability of the material through the Clausius–Mossotti relation. The large number of peaks for the BeP_2_ monolayer describe the dipole-allowed transition between states near the Van Hove singularities (VHS).^55^ According to the literature, materials with a bandgap below 1.7 eV function outstandingly in the IR region as optical materials.^56^

As shown in Fig. 6(a), based on the real part of the dielectric function, we analysed the static dielectric constant at the zero frequency limits, which is 34.49, 22.37, 15.20, 6.64 and 4.82 for the 0%, 2%, 4%, 6% and 8% BeP_2_ monolayer, respectively, suggesting that the 0% (strain-free) BeP_2_ monolayer has relatively high polarizability. Also, the negative values indicate the metallic nature of the 0% (strain-free) BeP_2_ monolayer in the IR region. There are various peaks in the imaginary part of the dielectric function, as shown in Fig. 6(b), which is due to the electronic transitions from the 3s/3p states in the valence band to the hybridized 2p orbitals of the Be atoms and 3s/3p orbitals of the P atoms, while the last peak is due to the electronic transitions from the 2p to 3s/3p orbitals. Here, the lower and broad peaks for the dielectric properties describe the volume plasmon oscillations and the absorption of plasmon energy, respectively. As shown in Fig. 6, it can be seen that the main loss in the optical curves occurs in the range of 8–7 eV and 8.5–9.5 eV, which is due to the oscillation of the electric dipoles and the ionizing environment.

**Fig. 7:**
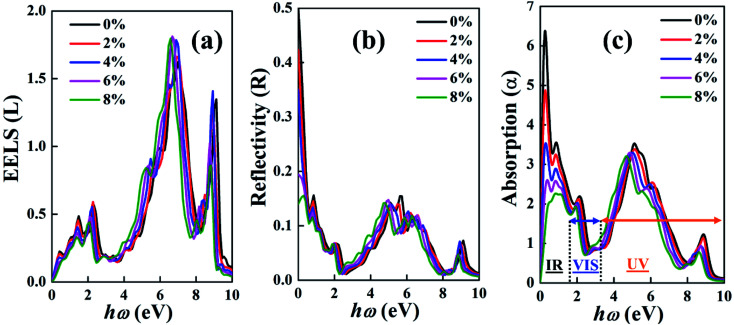
Calculated (a) electron energy loss spectrum (EELS) (*L*), (b) reflectivity (*R*) and (c) absorption spectra (*α*) of the dielectric function of the BeP_2_ monolayer with various Lagrangian strain.

Fig. 7(a) shows the good optical efficiency in the IR range based on the low values in the EELS curve. Fig. 7(b) and (c) present the reflectivity and absorption spectra of the BeP_2_ monolayer for various Lagrangian strain. Here, the interband transition can be understood based on the absorption and reflection spectra of the BeP_2_ monolayer, while the minimum value of the reflection and absorption is observed at 2.3 eV due to the volume plasmon oscillation from 2 to 3 eV with a gap of 1 eV. Here, the absorption coefficient was calculated as *A*(*ω*) = 2*ωK*(*ω*)/*C*, where, *K* is the extinction coefficient. The maximum absorption peaks occur due to the high extinction coefficient and minimum wave transfer. According to the reflectivity in Fig. 7(b), the BeP_2_ monolayer is a good electromagnetic wave absorber in the absorption range of around 0 eV to 9 eV. In this absorption range, several peaks are good reflectors of UV and VIS. Table 2 presents the values of the absorption and reflection peaks and the static reflection coefficient, *R*(0), based on Fig. 7(b) and (c).

**Table 2:** Maximum absorption peaks of the various BeP_2_ monolayers with various Lagrangian strain

Strain (%)	0	2	4	6	8
Absorption peaks (eV)	2.26, 5.16, 8.81	2.17, 5.16, 8.80	2.02, 5.04, 8.72	1.98, 4.92, 8.76	1.94, 4.69, 8.61
Reflection peaks (eV)	0.86, 2.13, 5.69, 9.13	0.78, 2.10, 5.57, 8.98	0.74, 2.04, 5.08, 8.89	0.74, 2.0, 4.96, 8.89	0.74, 1.98, 4.78, 8.80
*R*(0)	0.50	0.42	0.35	0.19	0.14

By analyzing the high absorption and low EELS up to 4 eV, we can conclude that the BeP_2_ monolayer is an optical absorber in the IR region. The IR region has many classification names such as near IR (NIR), short-wave IR (SWIR), mid-wave IR (MWIR), and long-wave IR (LWIR) region. Due to its electric charge oscillation and ionic behavior, the maximum reflection coefficient occurs near 5.5 eV.

## Conclusions

In conclusion, we used *ab initio* density functional theory to study the strain-dependent structural, electronic, and optical properties of the BeP_2_ monolayer. Accordingly, it was concluded that the stability of this system was confirmed by its cohesive energy such as 7.62, 3.48, 5.32, 3.94, 3.55 and 4.26 eV per atom and internal pressure. Upon applying tensile Lagrangian strain, the system became semiconductor in nature with direct band gaps in the range of 0.06 to 0.30 eV. Based on the dielectric function, the BeP_2_ nanosheets are good absorbents of electromagnetic waves in the IR region. This study is important because the bandgap is a fundamental issue that can limit the use of the emerging 2D BeP_2_ in the future. Thus, results present for the BeP_2_ nanosheets demonstrate their promising practical applications in novel mechanical–electronic devices and optical instruments such as night vision devices, thermo-graphic cameras, and infrared vibrational spectroscopy.

## Conflicts of interest

There are no conflicts to declare.

## Supplementary Material
